# Primary hip or knee arthroplasty in the setting of chronic suppressive antibiotics for prior periprosthetic joint infection: a scoping review

**DOI:** 10.5194/jbji-11-331-2026

**Published:** 2026-06-05

**Authors:** Vincent K. Melemai, Ryan J. Blake, Christian Barill, Adam E. Klein, Matthew J. Dietz, Allison M. Lastinger

**Affiliations:** 1 Department of Orthopaedics, West Virginia University, Morgantown, 26506, USA; 2 Department of Medicine, West Virginia University, Morgantown, 26506, USA

## Abstract

**Background:** Total joint arthroplasty (TJA) is an increasingly common intervention in the US, with an expected proportionate rise in periprosthetic joint infection (PJI). As part of the treatment plan, long-term suppressive oral antibiotic therapy is sometimes used in patients who would not benefit from further surgical intervention of their PJI. The aim of this scoping review is to outline the current evidence surrounding PJI incidence in new primary arthroplasty in this patient population. **Methods:** A search was conducted using the PubMed and Scopus databases with no date restrictions. Studies examining the incidence of developing a PJI after new primary hip or knee arthroplasty in patients receiving chronic suppressive antibiotic therapy for a prior PJI were included. Two authors independently screened the results for inclusion in this review. **Results:** Three retrospective cohort studies were ultimately included. Across studies, 61 patients with prior PJI receiving chronic suppressive antibiotics undergoing new primary TJA were identified. PJI rates in suppressed patients ranged from 0 % to 21.4 % compared to 0 % to 2.9 % in non-suppressed patients with prior PJI and 0 % to 2.6 % in matched controls without prior PJI. **Conclusion:** Limited evidence exists regarding PJI risk in new primary arthroplasty among patients on chronic suppression for prior PJI, with a wide range of incidence reported across studies. Due to a lack of direct evidence examining this specific patient population, further research must be conducted in order to support strong recommendations.

## 1 Introduction

Total joint arthroplasty has become the standard of care for treating late-stage hip and knee arthritis, providing excellent results in pain management and the return of function. Furthermore, total hip arthroplasty (THA) and total knee arthroplasty (TKA) continue to increase in the US, with a 67.9 % increase in THA and TKA from 2013 to 2022 (Jones et al., 2025). Orthopedic surgeons must be prepared to treat the proportionate rise in periprosthetic joint infection (PJI) as the current literature suggests rates of PJI in primary hip and knee arthroplasty to be between 0.3 % and 1.9 %, and upwards of 10 % in revision cases (Dobson and Reed, 2020). In the course of treatment for PJI, in addition to intravenous antibiotic therapy and revision surgical procedures, many patients are placed on long-term suppressive oral antibiotic therapy in cases where infection recurrence is likely or medical comorbidities preclude surgical eradication (Aboltins et al., 2025).

After achieving acceptable outcomes on long-term suppressive antibiotic therapy for treatment of a PJI, many patients may then seek primary arthroplasty for a different joint, with studies finding that up to 45 % of patients receive a second primary joint arthroplasty after their initial primary hip or knee replacement (Schaffler et al., 2025). In patients with prior PJI on suppressive antibiotic therapy, the request to proceed with a primary THA or TKA may pose a significant clinical challenge for both the orthopedic surgeon and the infectious diseases team to manage the existing PJI while simultaneously preventing PJI in the new primary arthroplasty.

Due to the limited evidence presented in the literature surrounding the risk of PJI in primary joints in patients receiving long-term suppressive antibiotic therapy for existing PJI, a scoping review was conducted to (1) systematically identify published evidence on PJI risk in new primary hip or knee arthroplasty among patients receiving chronic antibiotic suppression for prior PJI, (2) to characterize infection incidence and causative organisms in this population, and (3) to synthesize relevant evidence on risk stratification and prevention strategies to inform shared clinical decision-making.

## 2 Methods

A scoping review was conducted to identify relevant studies concerning primary arthroplasty in the setting of chronic antibiotic suppressive therapy for prior PJI. A literature search was conducted using the PubMed and Scopus databases.

The following Boolean phrase was used to search PubMed: “prosthetic joint infection” (tiab) OR “periprosthetic joint infection” (tiab) OR “peri-prosthetic joint infection” (tiab) OR PJI (tiab) AND antibiotic* (tiab) AND chronic (tiab) OR long-term (tiab) OR suppress* (tiab) AND “primary arthroplasty” (tiab) OR primary tiab) AND THA (tiab) OR hip (tiab) OR TKA (tiab) OR knee (tiab) NOT shoulder (tiab) OR elbow (tiab) OR ankle (tiab) AND English (lang).

To search Scopus, the following search phrase was used: TITLE-ABS-KEY “prosthetic joint infection” OR “periprosthetic joint infection” OR “peri-prosthetic joint infection” OR PJI AND antibiotic* AND chronic OR “long-term” OR suppressive AND “primary arthroplasty” OR primary OR arthroplasty AND NOT TITLE-ABS-KEY (shoulder OR elbow OR ankle).

For the purposes of this review, studies included were cohort studies, case control studies, case series, randomized controlled trials, systematic reviews, clinical practice guidelines, and consensus statements examining patients aged 18 years or older who had undergone a primary THA or TKA with a history of PJI in a different joint and who were receiving chronic antibiotic therapy at the time of new arthroplasty. Studies involving primary arthroplasty of the joints other than the hip or knee, animal studies, and articles not available in English were excluded.

**Figure 1:**
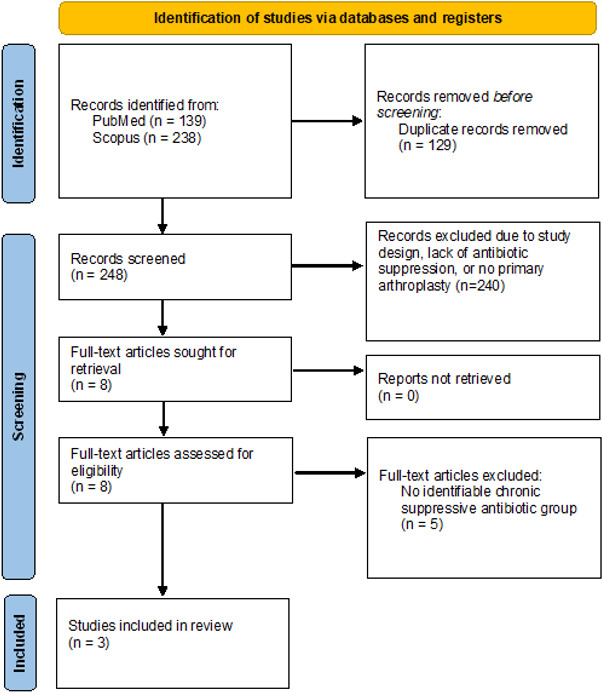
PRISMA flow diagram. Literature search, 16 December 2025.

The search was conducted on 16 December 2025, without date limitations in the search criteria. After duplicate removal, titles and abstracts were independently screened by two reviewers using Rayyan software (Cambridge, MA), with disagreements resolved through discussion or third-party adjudication. Inter-rater agreement was almost perfect ( κ=0.86 ). Full-text articles were similarly reviewed, and articles meeting inclusion criteria were selected for this scoping review (Fig. 1).

Data extraction was conducted for all included studies. Descriptive statistics were used to summarize the data, and data were extracted from each study into a standardized table (Tables 1, S1 in the Supplement). The data of interest for this review were study characteristics, patient demographics, type of arthroplasty, antibiotic therapy used, duration of therapy, follow-up duration, and identified PJI risk factors. Cumulative incidence was calculated as the number of PJI events divided by the number of patients in each subgroup.

**Table 1:** Studies examining PJI risk in new primary joints in the setting of suppressive antibiotic therapy for previous PJI. Three studies (2019–2022) from US centers included 61 patients with prior PJI receiving chronic suppressive antibiotics undergoing new primary THA or TKA. PJI rates in suppressed patients ranged from 0 % to 21.4 % compared to 0 % to 2.9 % in non-suppressed patients with prior PJI and 0 % to 2.6 % in matched controls without prior PJI. Fisher's exact test was performed in the SAT versus No SAT subgroup analysis. Abbreviations: PJI – periprosthetic joint infection, TKA – total knee arthroplasty, THA – total hip arthroplasty, TJA – total joint arthroplasty, MSIS – MusculoSkeletal Infection Society, SAT – suppressive antibiotic therapy. ^*^ No group with prior PJI without chronic suppressive antibiotics was identified. Comparison made with controls.

Subgroup analysis														
Author	Date range	Study design	PJI criteria	Total cohort	Study cohort	Matched control cohort	Control PJI incidence	SAT n | % of study cohort	SAT PJI incidence	No SAT n | % of study cohort	No SAT PJI incidence	Mean follow-up	Mean time from PJI to new primary	Fisher's exact test
Chalmers et al. (2019)	2000–2014	Case control	2011 MSIS	102 TKAs (95 patients)	102 TKAs (95 patients)	306 TKAs (306 patients)	2.6 % ( n=8 )	26 patients (28 TKAs) | 27 %	21.4 % ( n=6 )	69 patients | 73 %	1.4 % ( n=1 )	6 years	8 years	p=0.001
Chalmers et al. (2020)	2000–2014	Case control	Prior operative intervention	200 THAs (198 patients)	50 THAs (48 patients)	150 THAs (150 patients)	1.3 % ( n=2 )	13 patients | 27 %	0 %	35 patients | 73 %	2.9 % ( n=1 )	6 years	4 years	p=1
Humphrey et al. (2022)	2000–2021	Case control	Surgeon judgment 2011 MSIS 2018 MSIS	225 TJAs	45 TJAs	180 TJAs	0 %	22 patients | 49 %	4.5 % ( n=1 )	88 patients^*^	0 %^*^	4.10 years	Not specified	p=0.2

## 3 Results

The search of PubMed and Scopus yielded 377 results. After removing 129 duplicates, 248 records were reviewed. The title and abstract screening excluded 240 records. Eight records underwent full-text review, of which three were included in this scoping review. All included studies were published between 2019 and 2022, all published from centers in the US. The PRISMA flowchart is demonstrated in Fig. 1. The characteristics of the included studies are outlined in Table 1. Additional study characteristics and data are outlined in Table S1.

### 3.1 Study cohorts

All three studies examined the incidence of PJI in patients undergoing new primary arthroplasty with a history of PJI in another joint. In one study (Humphrey et al., 2022), the group examined the incidence of PJI in patients who were actively receiving suppressive antibiotics for any infectious process during a new primary arthroplasty from 2000 to 2021, including a sub-analysis of patients on chronic suppression for prior PJI, which was the group of interest for the purposes of this review. Due to the inclusion criteria set by Humphrey et al. (2022), no cohort of patients with a prior PJI without chronic suppression was identified. The other two studies (Chalmers et al., 2019, 2020) included patients on suppressive antibiotics for prior PJI at the time of new primary arthroplasty from 2000 to 2014 at a single institution and completed statistical analysis on this group; however, these two studies did not set suppressive antibiotic therapy as inclusion criteria. Instead, the study cohorts were patients with a prior PJI in another joint, which also included patients on chronic suppression (Table 1).

### 3.2 Definition of PJI

In 2011, the MusculoSkeletal Infection Society (MSIS) published a standardized set of diagnostic criteria for PJI (Parvizi et al., 2011), with a recommended update published in 2018 by Parvizi et al. which incorporated additional emerging diagnostic tests.

In the study by Chalmers et al. (2019), the 2011 MSIS criteria for PJI were used to define a history of PJI in the study population, while the study by Chalmers et al. (2020) used prior surgical intervention for PJI to define a history of PJI. The study by Humphrey et al. (2022) used the 2011 MSIS criteria for patients diagnosed between 2011 and 2018, and the updated 2018 criteria were used for patients diagnosed after 2018; those diagnosed with PJI before 2011 were based on best judgment from an orthopedic surgery and infectious disease team.

**Table 2:** Gap analysis of current evidence of primary arthroplasty in the setting of chronic antibiotic suppression.

Known	– Prior PJI represents a risk factor for metachronous infection – Some patients on suppression develop new-joint PJI – Organism concordance is variable (0 %–83 %)
Unknown	– Optimal antibiotic regimen for suppression during new primary arthroplasty – Duration of pre-operative suppression needed – Necessity of peri-operative suppression – Impact of specific organism types on outcomes – Role of patient comorbidities in risk stratification – Cost-effectiveness of proceeding with surgery vs. treating existing PJI first
Future research	– Multi-center prospective cohort studies – Standardized reporting of antibiotic regimens – Patient-reported outcomes and quality of life considerations – Decision analysis modeling

### 3.3 Incidence of PJI in the setting of suppressive antibiotics

The study by Chalmers et al. (2019) identified 26 patients with 28 TKAs (27 % of the 102 TKAs examined) as having been on chronic antibiotic suppression for prior PJI at the time of primary arthroplasty. In this study, the PJI incidence in patients on chronic suppression was significantly higher than those not on chronic suppression (HR 15.2; 95 % CI 8.4–23.2; p=0.002 ), with an incidence of 21.4 % ( n=6 ) in the chronic suppression group compared to 1.4 % ( n=1 ) in the group not on chronic suppression (Chalmers et al., 2019). In another study, Chalmers et al. (2020) examined 48 patients (50 THAs) who underwent primary THA, with 13 patients (27 % of the study group) on chronic suppression at the time of new primary THA. None of the 13 suppressed patients (0 %) developed PJI in the new THA compared to the development of one PJI in the 35 patients (2.9 %) who were not on chronic suppression, although this finding was not statistically significant. Lastly, in their sub-analysis examining 22 patients on chronic suppression for PJI, Humphrey et al. (2022) found only a 4.5 % ( n=1 ) incidence of PJI in a new primary arthroplasty compared to the control cohort ( n=88 ) incidence of 0 %, although this result was also not statistically significant ( p=0.2 ) (Table 1).

### 3.4 Organisms isolated in PJI

Only one of the three included studies described the causative organism for the PJI experienced in the new primary arthroplasty while on chronic antibiotic suppression. In their study examining new primary TKA, Chalmers et al. (2019) found that five of the six patients (83 %) on chronic suppression were infected with a different organism than the organism that caused their previous PJI.

## 4 Discussion

This scoping review identified only three studies examining PJI incidence while on chronic antibiotic suppression for a past PJI with high variability of reported infection rates (0 %–21 %). The heterogeneity of these studies combined with the small sample sizes (13–26 patients per study) preclude definitive conclusions about the safety of proceeding with new primary arthroplasty in this population.

### 4.1 Sources of heterogeneity and study limitations

The marked heterogeneity in PJI incidence across the included studies (0 %–21 %) likely reflects multiple factors beyond chance. Chalmers et al. (2019) reported the highest infection incidence (21 %) in TKA, but all three studies used similar PJI diagnostic criteria, suggesting that differences in patient selection, suppressive antibiotic regimens, organism types, or unmeasured confounders may explain outcome variability. None of the included studies reported specific antibiotic agents, dosing, or duration of suppression, precluding analysis of whether the treatment regimen influenced outcomes. Additionally, differences in surgeon decision-making regarding which patients are offered new primary arthroplasty while on suppression (selection bias) could substantially influence observed infection rates. Future studies must standardize the reporting of these critical variables to enable meaningful synthesis.

### 4.2 Practical implications for treatment strategies

The conflicting evidence on infection risk reported across studies in this review does not allow for strong recommendations to be made either in favor of or against new primary arthroplasty in the setting of chronic antibiotic suppression. However, clinicians can extrapolate potential risk from other studies. Bedair et al. (2015) found a history of PJI in another joint to be the sole risk factor for an increase in risk for PJI in a new primary arthroplasty, even in the absence of chronic antibiotic suppression. A study by Escudero-Sanchez et al. (2020) found that up to 25 % of patients on chronic antibiotic suppression may experience treatment failure between 2 and 5 years after initiation of suppression. Additionally, several studies have demonstrated likely hematogenous spread from prior PJI due to microorganism concordance between metachronous PJI in different joints, with a reported concordance rate up to 88.9 % (Hecht et al., 2025; Lee et al., 2021). Therefore, despite chronic antibiotic suppression, the potential risk of PJI in a new primary arthroplasty secondary to failure of antibiotic suppression must be communicated to patients.

### 4.3 Gap analysis

While evidence regarding this patient population is limited, identification of the current knowledge gaps is crucial to not only inform current practice but also to guide future research in this unique population. The current evidence, unknown variables, and recommendations for future research are outlined in Table 2. 

## 5 Conclusions

The limited available evidence provides conflicting findings for PJI risk in new primary arthroplasty among patients on chronic suppression for prior PJI. While one study reported substantially elevated risk (21 % PJI incidence in TKA), two others found low or no infections in suppressed patients. All studies were limited by variability in the outcomes observed, heterogenous cohorts, incomplete reporting of antibiotic suppressive regimens, small sample sizes, and a lack of statistical power. Therefore, further research must be conducted in order to provide strong treatment recommendations for this patient population.

## Supplement

10.5194/jbji-11-331-2026-supplement The supplement related to this article is available online at https://doi.org/10.5194/jbji-11-331-2026-supplement.

## Data Availability

The data used to support the findings of this article are outlined in Tables 1 and S1 and are publicly available.

## References

[bib1.bib1] Aboltins C, Lemoh C, Suleiman M, Soriano A, Davis J, Manning L (2025). Outcomes after suppressive antimicrobial therapy for prosthetic joint infection: a prospective cohort study. Antimicrob Agents Chemother.

[bib1.bib2] Bedair H, Goyal N, Dietz MJ, Urish K, Hansen V, Manrique J, Hamilton W, Deirmengian G (2015). A History of Treated Periprosthetic Joint Infection Increases the Risk of Subsequent Different Site Infection. Clin Orthop Relat Res.

[bib1.bib3] Chalmers BP, Weston JT, Osmon DR, Hanssen AD, Berry DJ, Abdel MP (2019). Prior hip or knee prosthetic joint infection in another joint increases risk three-fold of prosthetic joint infection after primary total knee arthroplasty: a matched control study. Bone Joint J.

[bib1.bib4] Chalmers BP, Berbari EF, Osmon DR, Hanssen AD, Berry DJ, Abdel MP (2020). Elevated Infection and Complication Rates in Patients Undergoing a Primary THA With a History of a PJI in a Prior Hip or Knee Arthroplasty: A Matched Cohort Study. J Arthroplasty.

[bib1.bib5] Dobson PF, Reed MR (2020). Prevention of infection in primary THA and TKA. EFORT Open Rev.

[bib1.bib6] Escudero-Sanchez R, Senneville E, Digumber M, Soriano A, Del Toro MD, Bahamonde A, Del Pozo JL, Guio L, Murillo O, Rico A, García-País MJ, Rodríguez-Pardo D, Iribarren JA, Fernández M, Benito N, Fresco G, Muriel A, Ariza J, Cobo J (2020). Suppressive antibiotic therapy in prosthetic joint infections: a multicentre cohort study. Clin Microbiol Infect.

[bib1.bib7] Hecht CJ, Ong CB, Arnold KE, Acuna AJ, Kamath AF (2025). Risk Factors, Diagnoses, and Clinical Characteristics of Synchronous and Metachronous Periprosthetic Joint Infections Following Total Joint Arthroplasty. J Arthroplasty.

[bib1.bib8] Humphrey TJ, Dunahoe JA, Nelson SB, Katakam A, Park ABK, Heng M, Bedair HS, Melnic CM (2022). Peri-Prosthetic Joint Infection in Patients Prescribed Suppressive Antibiotic Therapy Undergoing Primary Total Joint Arthroplasty: A 1:4 Case Control Matched Study. Surg Infect (Larchmt).

[bib1.bib9] Jones CM, Potluri AS, Federico VP, Nie JW, Forlenza EM, Serino 3rd J, Della Valle CJ (2025). Trends in Medicare Arthroplasty Procedure Volume: Projecting From 2025 to 2040. J Arthroplasty.

[bib1.bib10] Lee SH, Chang CH, Hu CC, Chang Y, Hsieh PH, Lin YC (2021). The Risk Factor and Outcome of Metachronous Periprosthetic Joint Infections: A Retrospective Analysis With a Minimum Ten-Year Follow-Up. J Arthroplasty.

[bib1.bib11] Parvizi J, Zmistowski B, Berbari EF, Bauer TW, Springer BD, Della Valle CJ, Garvin KL, Mont MA, Wongworawat MD, Zalavras CG (2011). New definition for periprosthetic joint infection: from the Workgroup of the Musculoskeletal Infection Society. Clin Orthop Relat Res.

[bib1.bib12] Parvizi J, Tan TL, Goswami K, Higuera C, Della Valle C, Chen AF, Shohat N (2018). The 2018 Definition of Periprosthetic Hip and Knee Infection: An Evidence-Based and Validated Criteria. J Arthroplasty.

[bib1.bib13] Schaffler BC, Prinos A, Kennedy MF, Ehlers M, Rozell JC, Schwarzkopf R (2025). Multiple primary joint arthroplasties and the risk of periprosthetic joint infection: evidence from a large retrospective cohort. J Arthroplasty.

