# Supplementary material for: Primary hip or knee arthroplasty in the setting of chronic suppressive antibiotics for prior periprosthetic joint infection: a scoping review
Source: J Bone Jt Infect. 2026 Jun 5;11(3):331–6. doi: 10.5194/jbji-11-331-2026 (PMC13238285; doi:10.5194/jbji-11-331-2026)
Supplement: The supplement related to this article is available online at https://doi.org/10.5194/jbji-11-331-2026-supplement. [file jbji-11-331-2026-supplement.pdf]

Supplement of J. Bone Joint Infect., 11, 331–336, 2026
https://doi.org/10.5194/jbji-11-331-2026-supplement
© Author(s) 2026. CC BY 4.0 License.

JBJI
Journal of Bone and Joint Infection
Open Access

Supplement of

Primary hip or knee arthroplasty in the setting of chronic suppressive antibiotics for prior periprosthetic joint infection: a scoping review

Vincent K. Melemai et al.

Correspondence to: Matthew J. Dietz (mdietz@hsc.wvu.edu)

The copyright of individual parts of the supplement might differ from the article licence.

Table S1

Table S1. Additional study characteristics

Author(s)	Study Group	Control Group	Follow-Up Duration	Time from PJI to New Primary
Chalmers et al. (2019)	History of: PJI in contralateral TKA or any THA; surgical treatment of PJI with component exchange; and receiving primary TKA	Matched for: age, sex, BMI, and date of index primary TKA	6 years (mean) (range: 2-16 years)	8 years (mean) (range: 4 months - 28 years)
Chalmers et al. (2020)	History of: PJI in contralateral THA or any TKA; surgical treatment of PJI with component exchange or resection arthroplasty; and receiving primary THA	Matched for: age, sex, BMI, and surgical year	6 years (mean) (range: 2-16 years)	4 years (mean) (range: 3 months - 18 years)
Humphrey et al. (2022)	History of: long-term oral (>6 months duration) SAT; maximum of 30 days SAT prior to TJA; and receiving new primary TJA	Matched for: age, sex, BMI, joint of primary THA or TKA, type 2 diabetes mellitus history, smoking history, and indication for primary TJA	4.10 years (mean) (± 3.42 years)	Not specified within study
